# #OrthoTwitter: Relationship Between Author Twitter Utilization and Academic Impact in Orthopaedic Surgery

**DOI:** 10.7759/cureus.33978

**Published:** 2023-01-19

**Authors:** Ryan T Halvorson, Sachin Allahabadi, Nicolas Cevallos, Aidan J Foley, Kelsey Collins, Abel Torres Espin, Brian T Feeley, Nirav K Pandya, Jeannie F Bailey

**Affiliations:** 1 Orthopaedic Surgery, University of California San Francisco, San Francisco, USA; 2 School of Medicine, University of California San Francisco, San Francisco, USA; 3 Orthopaedic Surgery, Washington University in St. Louis, St. Louis, USA; 4 Neurological Surgery, University of California San Francisco, San Francisco, USA; 5 Physical Therapy, University of Alberta, Edmonton, CAN

**Keywords:** orthopaedics, orthotwitter, twitter, orthopaedic research, orthopaedics surgery, author-level bibliometrics, bibliometric analyis, bibliometric analyses, ortho surgery, ortho

## Abstract

Background

#OrthoTwitter has evolved to disseminate findings and engage the public. However, the academic impact of Twitter utilization in orthopaedic surgery is unknown.

Questions/purposes

The purpose of the study was to evaluate relationships between the author and manuscript Twitter activity and citations.

Methods

Manuscripts in 17 orthopaedic journals from 2018 were identified. Citations, online mentions, impact factors, and subspecialties were obtained. H-index and Twitter account details for authors were obtained for a subset of manuscripts. Relationships between Twitter activity and citations were evaluated.

Results

2,473/4,224 (58.5%) manuscripts were mentioned on Twitter (n=29,958 mentions), with Twitter manuscripts cited more frequently (median 10 vs. 7, p<0.0001). Twitter mentions, impact factors, non-open-access status, and subspecialties were associated with citation counts. Articles mentioned in 10, 100, and 1,000 Tweets were observed to have a 1.1-fold, 1.7-fold, and 245-fold increase in citations. In author-level analyses, 156 (20.0%) first and 216 (27.7%) senior authors had Twitter accounts. Citation count was associated with increasing senior author H-index (*β*_est_=0.13, p<0.05), Twitter mentions (*β*_est_=0.0043, p<0.0001), impact factors (*β*_est_=0.13, p<0.0001), and having a first (*β*_est_=0.20, p<0.05) or senior author (*β*_est_=0.17, p<0.05) on Twitter. Articles published in arthroplasty (*β*_est_=0.49, p<0.05), general interest (*β*_est_=0.55, p<0.01), sports (*β*_est_=0.63, p<0.01), and non-open access journals (*β*_est_=0.41, p<0.001) were cited more. H-index correlated with followers for first (rho=0.31, p<0.0001) and senior authors (rho=0.44, p<0.0001).

Conclusion

Author Twitter utilization is independently associated with manuscript citations. Authors should be aware of the potential association between social media utilization and traditional academic impact. Understanding the relationship between social media utilization and academic impact is necessary to effectively disseminate research.

## Introduction

Social media is transforming the way academic medical research is shared. While articles are shared on a variety of platforms, more than four out of five online mentions of academic manuscripts occur on Twitter [1]. With over 400 million users and a brief character content sharing limit, Twitter offers a substantially greater audience and speed of dissemination compared to academic journals. Within the field of orthopaedic surgery, the "#OrthoTwitter" community on the Twitter platform has evolved as a forum to disseminate findings, exchange ideas, and connect with both the public and other practitioners [2-4]. It has been suggested that Twitter activity at the time of publication is associated with academic citation counts after several years [1,5,6]. However, the academic impact of social media utilization, particularly by authors, remains uncertain in the orthopaedic surgery community.

Orthopaedic surgery is also comprised of multiple diverse subspecialties - each with its own subset of journals and communities - which may influence the relationship between Twitter utilization and citations. While citation counts and online mentions have previously been reported for hand surgery [6] and sports medicine [5], cross-subspecialty comparative analyses are limited. It remains to be known how the relationship between online utilization and citations may vary among orthopaedic subspecialties.

Furthermore, while alternative metrics (e.g., Altmetric Attention Score [AAS]) have been proposed to modernize the estimated impact of manuscripts [7], there is no corresponding method of adjusting the impact of authors based on their individual online influence. Author-level productivity has historically been assessed using the Hirsch index, which is derived from the citation count of published manuscripts [8]. While authors maintain varying degrees of influence on social media, less is known about the relationship between online popularity and academic credibility and impact. For example, a recent analysis found 22 of the 100 most influential orthopaedic surgery Twitter accounts were not orthopaedic surgeons [9].

Understanding the association between social media utilization and academic impact and how this association may vary across subspecialties is increasingly necessary to effectively disseminate research and assess research impact. The purpose of the study was to evaluate the relationship between citation count and both manuscript-related Twitter activity and author Twitter utilization among orthopaedic surgery subspecialties. We hypothesized that author Twitter utilization and manuscript-related Twitter activity would be associated with greater citation counts and that there would be variability among orthopaedic subspecialties.

## Materials and methods

Data collection

Because this study utilized publicly available data, no Institutional Review Board approval was sought. No funding was received for this research.

Article Selection

Seventeen high-impact orthopaedic surgery journals, representing a range of subspecialties, were selected. Open access status, subspecialty, and impact factor were recorded (Table 1). Articles published in these journals between January 1, 2018 and December 31, 2018 were identified using the easyPubMed R package (Vienna, Australia) to query PubMed (https://pubmed.ncbi.nlm.nih.gov/) [10]. The authors, titles, abstracts, and digital object identifiers (DOI) were extracted from the identified article metadata using the same package. Letters to the editor, editor's notes, responses, errata, and technique videos were excluded. Because the analysis was performed in January 2022, articles published in 2018 were selected to overcome citation lag [11,12]. Article citation counts were obtained using the rscopus R package (RStudio, Boston, MA) [13].

**Table 1:** The seventeen orthopaedic surgery journals selected for analysis

Journal name	Open access	Subspecialty
Journal of Bone and Joint Surgery (JBJS)	N	General
Clinical Orthopaedics and Related Research (CORR)	N	General
Journal of the American Academy of Orthopaedic Surgeons (JAAOS)	N	General
Journal of Orthopaedic Trauma (JOT)	N	Trauma
Journal of Pediatric Orthopaedics (JPO)	N	Pediatrics
Journal of Pediatric Orthopaedics – B (JPO-B)	Y	Pediatrics
Journal of Children’s Orthopaedics (JCO)	Y	Pediatrics
Journal of Hand Surgery (JHS)	N	Hand and Upper Extremity
Journal of Shoulder and Elbow Surgery (JSES)	N	Hand and Upper Extremity
American Journal of Sports Medicine (AJSM)	N	Sports Medicine
Arthroscopy	N	Sports Medicine
Orthopaedic Journal of Sports Medicine	Y	Sports Medicine
Journal of Arthroplasty	N	Arthroplasty
Arthroplasty Today	Y	Arthroplasty
Spine	N	Spine
Global Spine Journal	Y	Spine
Foot & Ankle International (FAI)	N	Foot and Ankle

Article Online Activity

Altmetrics (www.altmetric.com) is an online platform and service that queries various online sources, including Twitter, Facebook, and mainstream media, for article mentions according to the National Information Standards Organization Data Quality Code of Conduct. Altmetrics automatically collects Tweets, Retweets, and quoted Tweets that contain a direct link to manuscripts. Twitter likes are not included. Altmetrics.com was selected as a source because it has been shown to have superior reliability in identifying Tweets compared to alternate sources [14]. The *rAltmetric* R package was used to query the Altmetrics database for the AAS and online article mentions [15].

Calculation of Author Hirsch Index

Hirsch developed an author-level measure of research impact, termed the h-index, which, although biassed towards researchers with longer careers, is commonly reported in bibliometric analyses [8]. An author’s h-index is defined as the integer, h, such that the author has at least h publications with at least h citations each. The h-index was calculated for the first author and senior author of each publication.

Author-Level Twitter Information

Twitter account handles were recorded for the first and senior authors for a random subset of 20% of the included manuscripts. Senior authors were assumed to be the last authors listed on a given manuscript. The subset was selected by sorting the articles in a randomized order and selecting the top 20%. The sample size was selected to balance feasibility with statistical power. The Twitter API (Twitter, San Francisco, CA) was employed to obtain author-level account details from the author account handles, including the number of account followers, the number of accounts followed, the number of tweets, and the account date of creation.

Statistical analyses

Data were analyzed using R version 4.1.2 (Vienna, Australia) [16]. Normally distributed data are summarized using the mean and standard deviation. Skewed data are summarized using the median and interquartile range (25th and 75th percentiles). To assess the correlation between continuous variables, Spearman correlation coefficients were calculated. To assess relationships between multiple groups (e.g., subspecialties), a Kruskall-Wallis one-way analysis of variance was performed. The correlation coefficients were interpreted according to Landis and Koch as none (<0), slight (0.01-0.2), fair (0.21-0.4), moderate (0.41-0.6), substantial (0.61-0.8), and almost perfect (0.81-1) [17]. P-values less than 0.05 were considered significant. All tests of significance were two-tailed.

The relationship between citations and manuscript-level data was assessed for the full set of manuscripts. The relationship between citations and manuscript- and author-level data were assessed for the specified randomized subset of 20% of manuscripts. Negative binomial logistic regression models were generated via generalized linear modelling to assess the relationship between citation count and author- and manuscript-level data. Negative binomial logistic regression models were selected as citation counts were over-dispersed. P-values were generated using Wald tests. In this approach, the presented beta coefficients represent the expected change in the natural log of citation count for a given one-unit increase in the predictor. A forced entry approach to variable selection was employed because associations between citation count and h-index, open access status, journal impact factor, Twitter mentions, and subspecialty have previously been suggested or hypothesized [4,18,19]. For consistency of interpretation, the subspecialty with the fewest median manuscript citations was selected as a reference in multivariable models.

Finally, the relationship between citations and author Twitter account details was assessed for both first and senior authors. Twitter account details were compared between first and senior authors, including the association between H-index and Twitter followers. The ratio of Twitter followers to H-index was compared among subspecialties [20].

## Results

After exclusion criteria were applied, 4,224 manuscripts remained, which had been cited a total of 53,635 times. Of the articles, 62.1% were mentioned online, and 94.2% of these (58.5% of the total) were mentioned on Twitter (Table 2). Manuscripts were mentioned on Twitter 29,958 times, on Facebook 1,667 times, and in the mainstream media 1,468 times. Among the full set of manuscripts, there was a positive correlation between the number of academic citations and the number of Twitter mentions (rho = 0.25, p < 0.0001), Facebook mentions (rho = 0.13, p < 0.0001), mainstream media mentions (rho = 0.21, p < 0.0001), and AAS (rho = 0.31, p < 0.0001).

**Table 2:** Summary of articles included from January 1, 2018 through December 31, 2018 Categorical variables are reported as n (%) where the % is in reference to the column total. Continuous variables are reported as median (interquartile range), except journal impact factor which is reported as value (standard deviation). Boldface p-values denote statistical significance (p < 0.05).

Variable	Overall	Articles mentioned on Twitter	Articles not mentioned on Twitter	P-value
Number (%)	4224	2473 (58.5)	1751 (41.5)	
Online activity				
Mentioned online (%)	2621 (62.10)	2473 (100.0)	148 (8.45)	
Mentioned on Facebook (%)	1050 (24.86)	1001 (40.48)	49 (2.80)	
Mentioned on mainstream media	293 (6.94)	223 (9.01)	70 (3.99)	
Altmetric activity score	1 (0–4.35)	2.95 (1.25–8.5)	0 (0–0)	<0.0001
Subspecialty (%)				<0.0001
Arthroplasty	712	293 (41.2)	419 (58.8)	
Foot and ankle	225	61 (27.1)	164 (72.9)	
General	757	456 (60.2)	301 (39.8)	
Hand and upper extremities	705	495 (70.2)	210 (29.8)	
Paediatrics	213	56 (26.3)	157 (73.7)	
Spine	405	277 (68.4)	128 (31.6)	
Sports	983	713 (72.5)	270 (27.5)	
Trauma	224	122 (54.5)	102 (45.5)	
Journal impact factor (SD)	3.19 (1.25)	3.15 (1.22)	3.25 (1.30)	0.013
Open access status (%)				
Yes	650 (15.4)	384 (15.52)	266 (15.19)	
No	3574 (84.6)	2089 (84.48)	1485 (84.81)	
Citations	8 (4–16)	10 (5–19)	7 (3–13)	<0.0001

Manuscript-Level Analyses

**Figure 1:**
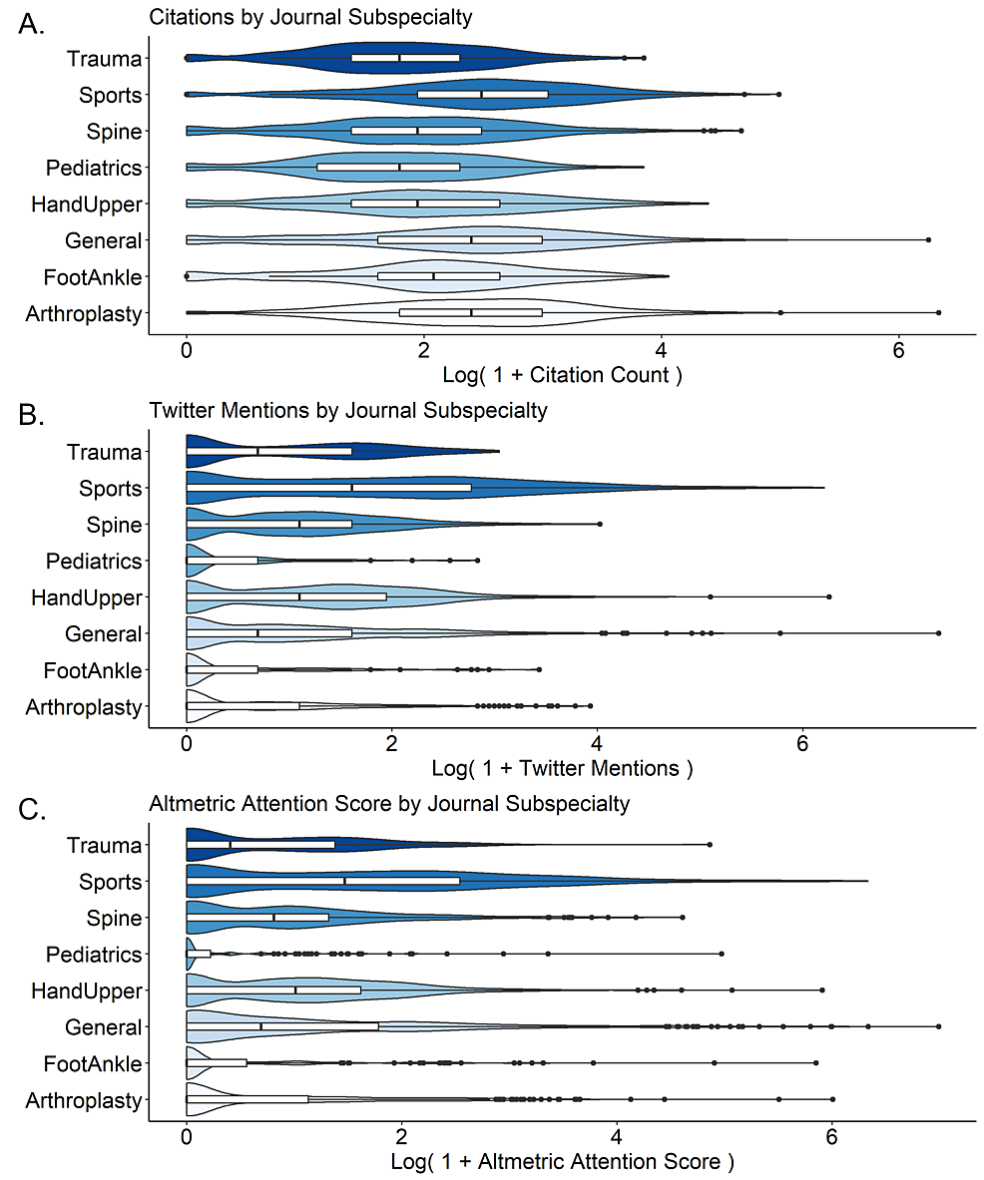
Manuscript citations (A), Twitter mentions (B), and Altmetric Attention Score (C) by subspecialty Data are presented as the natural logs of one plus citation count, Twitter mentions, and AAS respectively, in order to avoid taking the log of zero. Violin plots are scaled according to a Gaussian density approximation. Overlying boxplots depict median and interquartile range.

There were significant differences between subspecialties in both citations and Twitter mentions (Figure 1). Articles in sports medicine-related journals were cited the most frequently (median 11, IQR 6-20), while articles in paediatrics (median 5, IQR 2-9) and trauma (median 5, IQR 3-9)-related journals were cited least frequently (Figure 1). Similarly, articles published in sports medicine journals were mentioned on Twitter most frequently (72.5% of articles) and articles published in paediatrics journals were mentioned on Twitter least frequently (26.3% of articles, Figure 1). Articles published in open-access journals received fewer citations (median 9 vs. 6, p < 0.0001, Table 3).

**Table 3:** Relationship between article factors and citation count for all articles Data are reported as median (interquartile range). UE: upper extremity.

Citation count	Overall N = 4224	Articles mentioned on Twitter N = 2473	Articles not mentioned on Twitter N = 1751
Overall	8 (4–16)	10 (5–19)	7 (3–13)
Subspecialty			
Arthroplasty	10 (5–19)	11 (5–22)	9 (5–17)
Foot and ankle	7 (4–13)	10 (6–15)	6 (3–11.25)
General	10 (4–19)	11 (5–20)	8 (2–15)
Hand and upper extremities	6 (3–13)	7 (3–15)	6 (2–11)
Paediatrics	5 (2–9)	5 (2–8.25)	5 (2–9)
Spine	6 (3–11)	7 (3–14)	4 (2–7)
Sports	11 (6–20)	13 (7–24)	7.5 (3–13)
Trauma	5 (3–9)	6 (3–11)	4 (2–7)
Open access status			
No	9 (4–17)	10 (5–20)	7 (3–14)
Yes	6 (3–12)	8 (4–15)	5 (2–8.75)

In multivariable analysis over the full set of articles, the number of Twitter mentions (*β*_est_ = 0.0055, p < 0.0001), journal impact factor (*β*_est_ = 0.14, p < 0.0001), and non-open access status (*β*_est_ = 0.35, p < 0.0001) were associated with greater citation count. For purposes of interpretation, the log of the number of citations was observed to increase by 0.0055 with each additional tweet mentioning the article. For context, 10 tweets would be associated with a 1.1-fold increase in citations, 100 would be associated with a 1.7-fold increase, and 1,000 tweets would be associated with a 245-fold increase. Compared to articles published in pediatrics-related journals, articles published in all other subspecialty-related journals except trauma received more citations (Table 4).

**Table 4:** Negative binomial regression of all 4224 articles to assess the relationship between manuscript-level factors and citation count Null deviance 5447 on 4223 degrees of freedom. Residual deviance 4763 on 4213 degrees of freedom. Theta 1.23, standard error 0.0291.

Variable	*β*_est_	Std. error	P-value
Twitter mentions	0.00552	0.0004	<0.0001
Subspecialty			
Paediatrics	Reference	Reference	
Arthroplasty	0.484	0.087	<0.0001
Foot and ankle	0.256	0.937	<0.01
General	0.476	0.081	<0.0001
Hand and upper extremities	0.237	0.078	<0.01
Spine	0.294	0.093	<0.01
Sports	0.584	0.083	<0.0001
Trauma	−0.350	0.094	0.71
Journal impact factor	0.141	0.016	<0.0001
Open access status			
No	Reference	Reference	
Yes	−0.347	0.053	<0.0001

Author-Level Analyses

**Figure 2:**
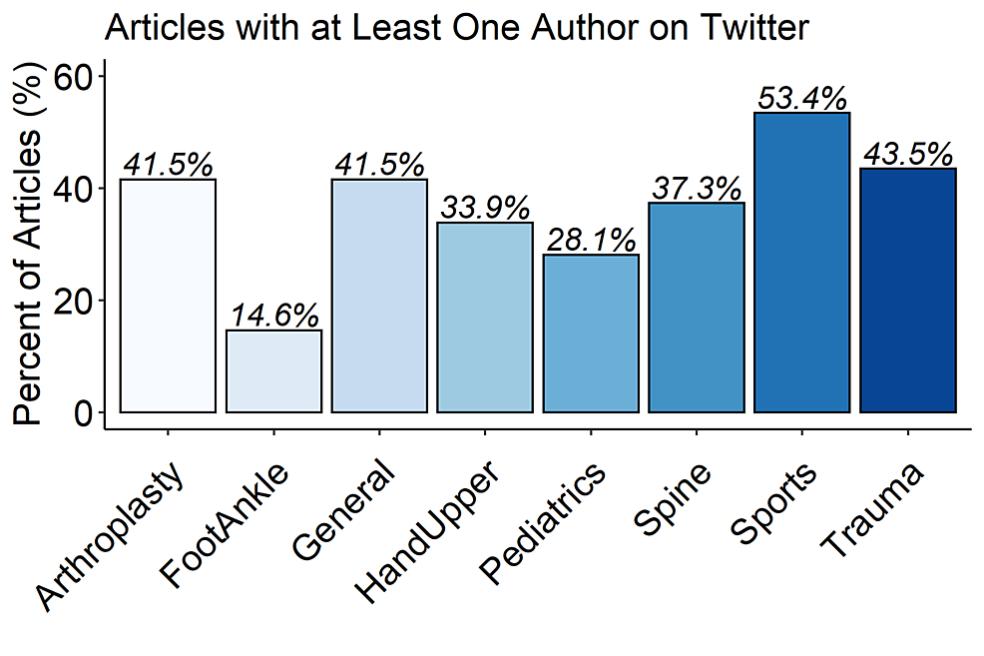
Percentage of articles having at least one of first or senior author with a Twitter account Overall Kruskal Wallis p-value is <0.0001.

**Figure 3:**
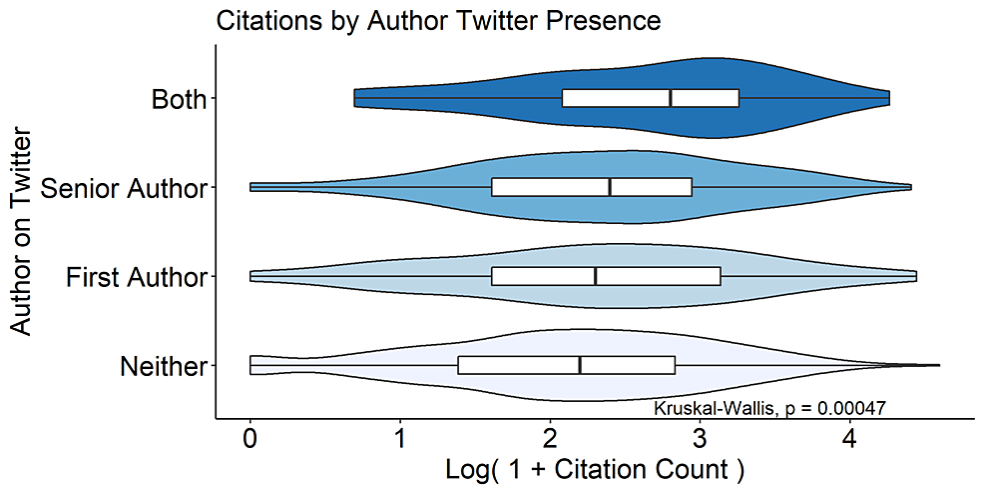
Manuscript citations by author Twitter presence Data are presented as the natural log of one plus citation count in order to avoid taking the log of zero. Violin plots are scaled according to a Gaussian density approximation. Overlying boxplots depict median and interquartile range.

A random selection of 20% of the manuscripts (n=780) was selected for author-level analyses after exclusion. Three hundred and twenty (41%) of these manuscripts featured either a first or senior author with a Twitter account, with 156 first-author accounts and 216 senior-author accounts (Table 5). There were significant differences in rates of author Twitter presence by journal subspecialty, with sports medicine-related journals featuring a Twitter author most frequently (53.4%) and foot and ankle-related journals least frequently (14.6%, Figure 2). Manuscripts with at least one author on Twitter were cited more frequently than those without either author on Twitter (median 10 vs. 8, p < 0.001, Figure 3). Articles with both first and senior authors on Twitter were cited most frequently (median 15.5, IQR 7-25).

**Table 5:** Summary of a random sample of 780 articles selected for author-level analyses Categorical variables are reported as n (%) where the % is in reference to the column total. Continuous data are reported as median (interquartile range). Boldface p-values denote statistical significance (p < 0.05). UE: upper extremity.

Variable	Overall	Both first and senior authors on Twitter	First author on Twitter	Senior author on Twitter	Neither the first nor the senior author	P-value
Number (%)	780	52 (6.7%)	104 (13.3%)	164 (21.0%)	460 (59.0%)	
Online mentions						
Twitter	1 (0–5)	8.5 (2–33.3)	2 (0–8)	1 (0–5)	1 (0–3)	
Facebook	0 (0–0)	0 (0–1)	0 (0–1)	0 (0–0)	0 (0–0)	
Mainstream media	0 (0–0)	0 (0–0)	0 (0–0)	0 (0–0)	0 (0–0)	
Altmetric attention score	1 (0–4.6)	6.9 (1.8–35.5)	1.4 (0–5.8)	1.2 (0–6.6)	0.5 (0–2.9)	<0.0001
Subspecialty (%)						<0.0001
Arthroplasty	142 (18.2%)	4 (2.8%)	20 (14.1%)	35 (24.6%)	83 (58.5%)	
Foot and ankle	41 (5.3%)	0 (0%)	2 (4.9%)	4 (9.8%)	35 (85.4%)	
General	142 (18.2%)	12 (8.5%)	25 (17.6%)	22 (15.5%)	83 (58.5%)	
Hand and UE	121 (15.5%)	5 (4.1%)	16 (13.2%)	20 (16.5%)	80 (66.1%)	
Paediatrics	32 (4.1%)	0 (0%)	5 (15.6%)	4 (12.5%)	23 (71.9%)	
Spine	67 (8.6%)	3 (4.5%)	10 (14.9%)	12 (17.9%)	42 (62.7%)	
Sports	189 (24.2%)	26 (13.8%)	20 (10.6%)	55 (29.1%)	88 (46.6%)	
Trauma	46 (5.9%)	2 (4.3%)	6 (13%)	12 (26.1%)	26 (56.5%)	
Journal impact factor	2.7 (2.2–4.6)	4.6 (2.4–5.0)	3.0 (2.2–4.6)	2.6 (2.4–4.6)	2.7 (2.2–4.6)	<0.0001
Open access status						<0.01
Yes	108	5	17	36	50	
No	672	47	87	128	410	
Citations	8 (4–17)	15.5 (7–25)	9 (4–22)	10 (4–18)	8 (3–16)	<0.001
First author (N = 156)						
H-Index	9 (4–17)	15.0 (7.0–19.5)	13.0 (7.25–19.75)	10.5 (5.0–16.0)	8.0 (4.0–16.0)	<0.001
Twitter followers	228.5 (60.8–752.5)	348 (109–1000)	196 (54–569)			
Twitter following	154.5 (55.0–424.0)	236 (97–489)	122 (53.8–362)			
Tweets	108.5 (22.8–474.0)	218 (39–611)	94 (21–461)			
Account age (years)	6.4 (4.1–11.1)	6.1 (4.1–10.4)	6.7 (4.2–11.1)			
Senior author (N = 216)						
H-Index	21 (12–36)**	36.0 (23.0–46.0)	21.0 (12.0–36.0)	28.0 (15.5–44.5)	19.0 (11.0–30.0)	<0.0001
Twitter followers	534.5 (125.5–1874)**	1065 (319–2636)		419 (104–1372)		
Twitter following	160 (51.0–475.5)	209 (62–576)		150 (48–444)		
Tweets	236.0 (46.3–971.0)*	352 (103–2076)		154 (36–738)		
Account age (years)	7.4 (5.2–11.2)*	6.8 (5.5–11.3)		7.5 (5.0–11.2)		

In multivariable analyses over this subset, a higher citation count was independently associated with a higher senior author H-index (*β*_est_ = 0.13, p < 0.05), number of Twitter mentions (*β*_est_ = 0.0043, p < 0.0001), journal impact factor (*β*_est_ = 0.13, p < 0.0001), and having a first (*β*_est_ = 0.20, p < 0.05) or senior author (*β*_est_ = 0.17, p < 0.05) with a Twitter account. Compared to pediatrics-related journals, articles published in arthroplasty (*β*_est_ = 0.49, p < 0.05), general interest (*β*_est_ = 0.55, p < 0.01), sports-related journals (*β*_est_ = 0.63, p < 0.01), as well as non-open access articles (*β*_est_ = 0.41, p < 0.001), were associated with more citations (Table 6). Because there was a very high (rho > 0.90) correlation between AAS and tweets, AAS was not included in the final model. Similar parameter estimates and significance levels were demonstrated in sensitivity analyses where AAS replaced the number of tweets.

**Table 6:** Negative binomial regression over the subset of articles to assess the association between manuscript and author-level factors and citation count Overall null deviance 1048 on 779 degrees of freedom. Residual deviance 878 on 764 degrees of freedom. Theta 1.416, standard error 0.080.

Variable	*β*_est_	Std. error	P-value
Twitter mentions	0.0043	0.0010	<0.0001
Author Twitter status			
Neither	Reference	Reference	
First author	0.204	0.098	<0.05
Senior author	0.167	0.085	<0.05
Both authors	0.062	0.136	0.65
H-index			
First author	0.0028	0.0025	0.272
Senior author	0.0036	0.0018	<0.05
Subspecialty			
Paediatrics	Reference	Reference	
Arthroplasty	0.494	0.202	<0.05
Foot and ankle	0.374	0.219	0.09
General	0.547	0.193	<0.01
Hand and UE	0.267	0.187	0.15
Spine	0.364	0.215	0.09
Sports	0.633	0.196	<0.001
Trauma	0.091	0.215	0.672
Journal impact factor	0.133	0.033	<0.0001
Open access status			
No	Reference	Reference	
Yes	−0.416	0.118	<0.001

Among the first authors, there were fair positive correlations between citations and the number of account followers (rho = 0.25, p < 0.01), the number of following accounts (rho = 0.29, p < 0.001), and the number of tweets (rho = 0.26, p < 0.01). Among senior authors, there was a slight positive correlation between citations and the number of account followers (rho = 0.16, p < 0.05), but there was no significant correlation between citations and tweets (rho = 0.12, p = 0.06) or following accounts (rho = 0.02, p = 0.80). There was also no relationship between account age and citations for either the first (rho = −0.01, p = 0.86) or senior (rho = 0.11, p = 0.1) authors.

**Figure 4:**
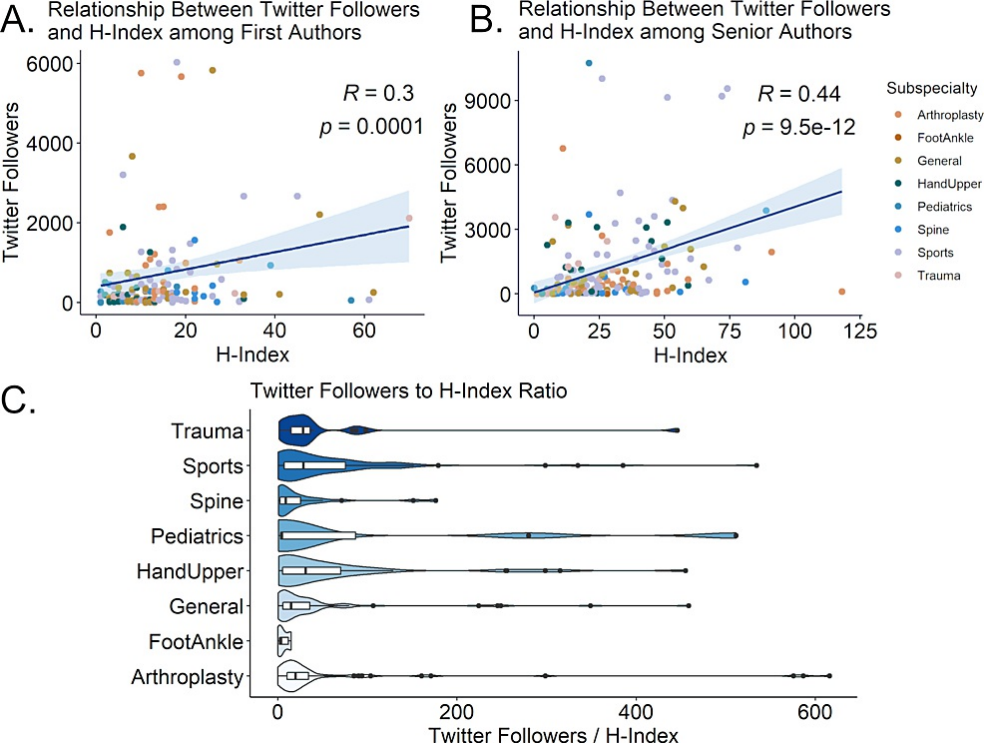
Relationship between Twitter followers and H-index among first and senior authors (A) The graph shows a scatterplot of H-index vs Twitter followers for first authors. (B) The graph shows a scatterplot of H-index vs Twitter followers for senior authors. (C) This chart represents the ratio of Twitter followers to H-index for manuscript authors by subspecialty. Violin plots are scaled according to a gaussian density approximation. Overlying boxplots represent median and interquartile range.

Senior authors had higher H-indices than the first authors (median 21 vs. 9, p < 0.0001). Senior author Twitter accounts had more followers (median 534.5 vs. 228.5, p < 0.0001), posted more tweets (median 236 vs. 108.5, p < 0.05), and had older Twitter accounts (median 7.4 vs. 6.4 years, p < 0.05). First and senior author accounts were following similar numbers of accounts (median 160 vs. 154.5, p = 0.86). The correlation between the H-index and Twitter followers was fair (rho = 0.31, p < 0.0001) for the first authors and moderate (rho = 0.44, p < 0.0001) for senior authors (Figure 4). There was significant variation by a subspecialty in the ratio of Twitter followers to H-index (Kruskal Wallis p < 0.05), with hand and upper extremity (median 31.0, IQR 5.29-70.16) and sports medicine having the highest ratios (median 28.75, IQR 7.0-75.8), foot and ankle (median 3.6, IQR 1.8-11.8), and paediatrics (median 5.7, IQR 3.6-280.0) having the lowest (Table 7).

**Table 7:** Twitter follower to H-index ratio among subspecialties Presented as median and interquartile range. IQR: interquartile range.

Subspecialty	Median (IQR)
Arthroplasty	20.0 (10.5–34.3)
Foot and ankle	3.6 (1.8–11.8)
General	14.8 (5.7–35.7)
Hand and upper extremity	31.0 (5.3–70.2)
Pediatrics	5.7 (3.6–280.0)
Spine	9.0 (2.5–25.6)
Sports	28.8 (7.0–75.6)
Trauma	28.2 (14.8–36.0)

## Discussion

Although only a quarter of authors had a Twitter account, those who did received more citations, even after controlling for subspecialty, journal impact factor, H-index, and open access status. There were significant differences by subspecialty in citations, Twitter mentions, author Twitter utilization, and the ratio of Twitter followers to H-index. This is the first study to consider author-level Twitter utilization in relation to both subspecialty and citation count and is also the largest study in the orthopaedic surgery literature on the topic of factors correlating with citations.

This study identified the author's Twitter presence as an independent predictor of increased citations. While the mean H-index of the top 72 most influential orthopaedic surgeon Twitter accounts has been reported to be 13.7 [9], the association between Twitter utilization and manuscript citations has not previously been described. Interestingly, Tweet count and Twitter follower count correlated with citations more strongly for first compared to senior authors, suggesting Twitter activity may be more strongly related to citation count for those earlier in their careers. Younger researchers, who have fewer publications, have likely attended fewer conferences, and likely have smaller professional networks, may rely more heavily on online platforms for networking than their more senior peers. In contrast, citations for senior authors were more closely related to academic metrics, such as H-index.

Regarding the influence of subspecialty, articles published in sports medicine, general interest, and arthroplasty journals were cited more, with sports medicine journal manuscripts being cited most (median 11, IQR 6-20). This corroborates a recent study that found arthroplasty, oncology, and sports medicine to be the three highest-cited orthopaedic subspecialties [21]. Additionally, sports medicine journals had the highest percentage of authors on Twitter (53.4% overall) and the highest percentage of manuscripts being mentioned on Twitter (72.1%). Factors contributing to the higher citation count may include the competitiveness of the specialty as well as the degree of specialization and the number of journals. Despite having the highest citation count, sports medicine-related authors also had one of the highest Twitter follower-to-H-index ratios, suggesting their accounts draw even more attention than would be expected for a given H-index. Sports medicine surgeons may be more likely to engage in online activity related to topics of popular interest (e.g., professional athlete injuries), which could increase their follower count. Arthroplasty-related journals may also receive more citations through a similar mechanism. It is expected that general interest journals (JBJS, JAAOS, and CORR) receive greater attention given their larger audience.

While Twitter previously accounted for 82% of the online article mentions in orthopaedics [1], the present study found that Twitter now accounts for over 90%, indicating the platform may be increasing its share of orthopaedic surgery-related social media utilization. Twitter utilization in orthopaedics may be similar to other medical specialties. Our estimate of 58.5% of articles mentioned on Twitter is lower than 73% in urology [22] but higher than 50.7% in otolaryngology [23]. Regarding effect size, urology and otolaryngology articles mentioned on Twitter received 2.0-fold and 1.6-fold more citations than those not mentioned, respectively. In contrast, the present study found a 1.3-fold increase in citations for articles mentioned on Twitter (median 10 vs. 7). Importantly, these authors approximated effect sizes using mean citation counts [22,23], which are highly skewed, and estimates based on the median would likely be lower.

The study corroborated the findings of prior studies by demonstrating positive correlations between Twitter mentions (rho = 0.25) and AAS (rho = 0.31) and citations. In 2017, Evanvew et al. analyzed the relationship between AAS and citations for 1,675 randomized controlled trials and estimated the rho to be 0.11 [1]. In 2020, Zhang and Earp estimated rho to be 0.10 for AAS and 0.11 for Twitter mentions for 835 articles from JAAOS, JBJS, and CORR [21]. Also in 2020, Kunze et al. estimated rho to be 0.31 for AAS in a sample of 496 articles from seven journals [18]. Reasons for the larger correlation in the present study compared to the prior studies include the larger sample of manuscripts from a range of journals and manuscript types, the increasing time between article publication and analysis, and the increasing utilization of Twitter. 

Interestingly, articles published in open-access journals received fewer citations, even when controlling for the impact factor. Prior literature on the impact of open access status has been conflicting. For example, Silva et al. identified a weakly positive association among sports medicine articles [5], but an inverse relationship has also been identified in other fields [19]. Overall, a weak effect of open access status and citations may be due to the prevalence of institutional availability of non-open-source articles. Furthermore, open-access journals are relatively young compared to other established subscription journals, which may contribute to lower citations or a lower preference for authors to cite articles from those journals.

The strengths of this article include the incorporation of author-level social media utilization, a large sample size of manuscripts, and the inclusion of a variety of journals representing different subspecialties. There are several limitations to our analysis of the author Twitter profiles, however. For example, some authors may run pseudonymous Twitter accounts that cannot be identified. The content of the author's tweets may also be unrelated to medicine. The exact mechanism of the Twitter feed-generating algorithm is not known and may bias the presentation of certain articles. Finally, an author's propensity for Twitter utilization may not be independent of other personal or professional factors related to academic productivity. Additionally, aside from applying exclusion criteria to certain types of articles, this study did not adjust for specific study design factors such as sample size or risk of bias. Finally, journal-specific Twitter activity and other factors may also be associated with citations [6,24]. Ultimately, it is not possible to determine causation in this retrospective analysis; it is not clear if articles mentioned on Twitter ultimately reach a higher impact as measured by citations or if higher-impact articles are more likely to be shared on social media. Other social media sectors not analyzed, such as Facebook, LinkedIn, and orthopaedic-specific platforms like AOC Connect and Ortho Social Media Platform, may also have impacts beyond Twitter and warrant future investigation to understand the ideal media for dispersing knowledge and research.

Author Twitter utilization is associated with academic citation count independently of other author and manuscript factors. Future studies should examine the content of tweets, the scientific quality of manuscripts, and the Twitter activity of specific journals. As research dissemination rapidly evolves online, authors should be aware of the impact of social media utilization on academic impact in order to effectively share findings and assess research impact.

## Conclusions

Twitter utilization and manuscript citations are independently associated with authors who publish in orthopaedic journals. Those who appreciate this potential link between social media implementation and traditional academic impact can benefit from more effective dissemination of their research. Additionally, understanding this association is essential for appraising the impact of evolving online platforms for research circulation. Future studies should examine Twitter activity within specific institutions and journals, the content of tweets, and the scientific quality of the manuscripts shared on the platform.
